# Resveratrol reduces store-operated Ca^2+^ entry and enhances the apoptosis of fibroblast-like synoviocytes in adjuvant arthritis rats model via targeting ORAI1–STIM1 complex

**DOI:** 10.1186/s40659-019-0250-7

**Published:** 2019-08-19

**Authors:** Jinsen Lu, Jiazhao Yang, Yongshun Zheng, Shiyuan Fang, Xiaoyu Chen

**Affiliations:** 1 Department of Orthopaedics, Anhui Provincial Hospital, Anhui Medical University, Lujiang Road No. 17, Hefei, 230001 Anhui People’s Republic of China; 2 Department of Histology and Embryology, Anhui Medical University, Meishan Road No. 81, Hefei, 230032 China

**Keywords:** Resveratrol, Apoptosis, Store-operated calcium entry, STIM1, ORAI1

## Abstract

**Background:**

Resveratrol was reported to trigger the apoptosis of fibroblast-like synoviocytes in adjuvant arthritis rats but the subcellular mechanism remains unclear. Since ER stress, mitochondrial dysfunction and oxidative stress were involved in the effects of resveratrol with imbalance of calcium bio-transmission, store operated calcium entry (SOCE), a novel intracellular calcium regulatory pathway, may also participate in this process.

**Results:**

In the present study, Resveratrol was found to suppress ORAI1 expression of a dose dependent manner while have no evident effects on STIM1 expressive level. Besides, resveratrol had no effects on ATP or TG induced calcium depletion but present partly dose-dependent suppression of SOCE. On the one hand, microinjection of ORAI1 overexpressed vector in sick toe partly counteracted the therapeutic effects of resveratrol on adjuvant arthritis and serum inflammatory cytokine including IL-1, IL-6, IL-8, IL-10 and TNF-α. On the other hand, ORAI1 SiRNA injection provided slight relief to adjuvant arthritis in rats. In addition, ORAI1 overexpression partly diminished the alleviation of hemogram abnormality induced by adjuvant arthritis after resveratrol treatment while ORAI1 knockdown presented mild resveratrol-like effect on hemogram in rats model.

**Conclusion:**

These results indicated that resveratrol reduced store-operated Ca^2+^ entry and enhanced the apoptosis of fibroblast-like synoviocytes in adjuvant arthritis rats model via targeting ORAI1–STIM1 complex, providing a theoretical basis for ORAI1 targeted therapy in future treatment with resveratrol on rheumatoid arthritis.
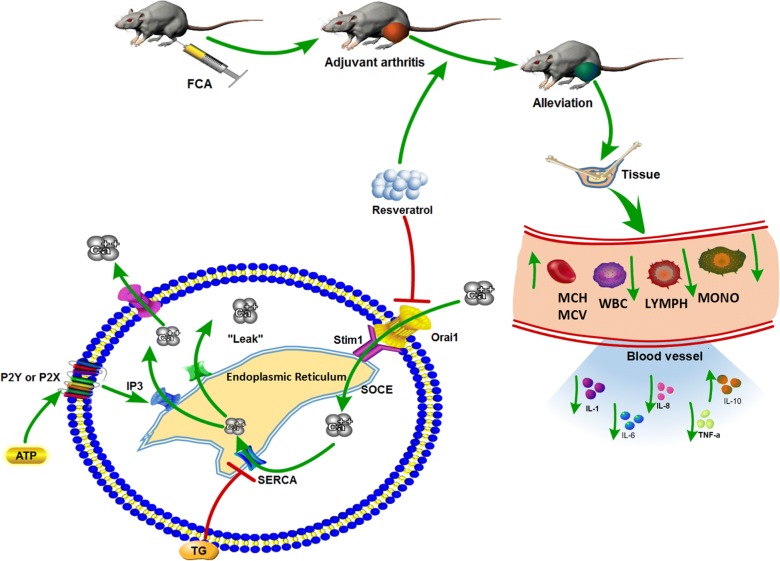

**Electronic supplementary material:**

The online version of this article (10.1186/s40659-019-0250-7) contains supplementary material, which is available to authorized users.

## Background

Resveratrol is a naturally active polyphenolic substance derived from plants such as peanuts and grapes. It has a wide range of benign biological effects including anti-inflammatory, anti-oxidation, cardiovascular protection and anti-cancer effects [1]. Besides, resveratrol has been shown to have therapeutic potential for autoimmune diseases such as rheumatoid arthritis, rheumatoid arthritis and systemic lupus erythematosus [2]. Our previous research has confirmed that resveratrol can inhibit the abnormal proliferation of fibroblast cells in AA rat models and induce apoptosis as well as autophagy [3]. In this pathological process, the imbalance of mitochondrial homeostasis, oxidation and antioxidant regulation plays an important role [4]. Moreover, we have accidentally discovered that the intracellular calcium homeostasis has been unbalanced during the study of cell mitochondria and oxidative stress changes after resveratrol treatment, but the specific regulation needs further study [5]. Store operated calcium entry (SOCE) is an intracellular biological process, in which a specific group of calcium channels mediate extracellular calcium influx for supplement after depletion of calcium store induced via outer stimuli [6, 7]. SOCE is widely involved in many physiological processes such as cellular immunity, tumor migration, muscle movement and blood coagulation [8, 9]. Store operated calcium channels (SOCs) are a class of non-voltage-dependent small but highly calcium-selective ion channels that can be activated by G proteins or a tyrosine kinase, phospholipase C, phosphatidylinositol 4,5-bisphosphate (PIP2) and inositol 1,4,5-trisphosphate (IP3) related signal transduction processes and participate in various disease processes [1]. STIM1 and ORAI1 are the first major mediators found in SOCE. During the process of SOCE, the emptying of calcium stores can be sensed by STIM1, and then STIM1 is transferred to the cell membrane to bind and activate ORAI1 to form a multimeric channel, which mediates the influx of extracellular calcium to supplements the loss of the calcium reservoir [10]. Deletion or abnormal expression of STIM1 and ORAI1 leads to congenital unprogressive myopathy, coagulopathy, T and B cell dysfunction and also affects tumor cell growth and invasion [11]. Since SOCs are widely distributed over various types of non-excitable cells, they are widely involved in the regulation of various biological functions [12]. As an important second messenger in the cell, calcium ion is a bridge connecting endoplasmic reticulum stress and mitochondrial dyshomeostasis [13]. The abnormal accumulation of intracellular calcium ions in time and space will determine the fate of the cell. Recent studies have shown that SOCs play an important role in the apoptosis process of various types of cells such as neuronal cells and hepatocarcinoma cells, and these effects are closely related to the regulation of STIM1, ORAI1 and their subtypes [14, 15]. However, there is no unified view on the regulatory direction of STIM1\ORAI1 expression and the corresponding biological effects. Overexpression of STIM1 and ORAI1 enhances the ability of some tumor cells to resist apoptosis, but leads to apoptosis of other tumor cells [16]. Therefore, the biological effects of STIM1 and ORAI1 should be closely related to different cell types and functions. Rheumatoid arthritis (RA) is a chronic autoimmune disease characterized by over-oxidative joint micro-environment and hyperplasia of synovial cells [17]. Inhibition of hyperproliferative synovial cells is a crucial point to alleviate joint symptoms of patients [18]. Our previous study has confirmed that resveratrol can alleviate serum lipid peroxidation, arthritis score and histopathological damage in rat AA model and induce apoptosis of rat fibroblast cells. Besides, based on our former study, 5 μM H_2_O_2_, rather than higher or lower dose, lead to slight proliferation of fibroblast in vitro, which provided a bio-model to imitate cytoproliferative and oxidative microenvironment in human RA enhancing the reliability of in vitro cell-based study [5]. Recent studies have shown that resveratrol can attenuate high glucose-induced apoptosis of vascular endothelial cells by regulating SOCE, suggesting that resveratrol may be involved in the regulation of specific apoptosis via SOCs [19]. Therefore, based on the previous research, our group further explored the specific mechanism of resveratrol-induced apoptosis in fibroblasts within 5 μM H_2_O_2_, and attempted to elucidate the regulation of resveratrol on SOCE and two essential SOCs, STIM1 and ORAI1. If there is some influence, whether the reverse adjustment of the effect can reverse the original effect need to be explored.

## Results

### Resveratrol reduces ATP or TG induced SOCE in FLSs

**Fig. 1:**
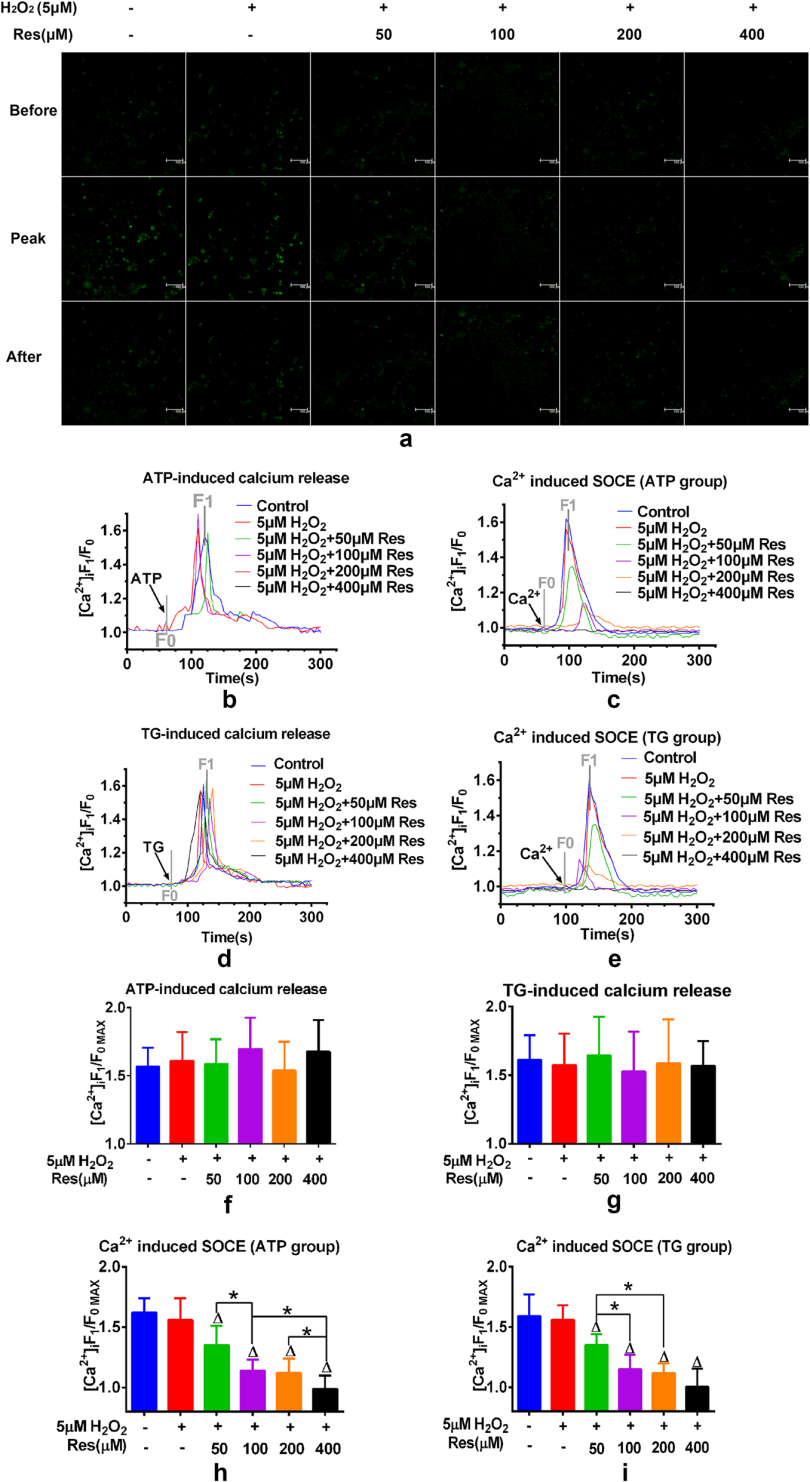
Resveratrol reduces ATP or TG induced SOCE in FLSs. FLSs were treated with different dose of resveratrol under 5H_2_O_2_ and ATP or TG induced calcium depletion combined with subsequent SOCE induced via extracellular calcium were detected via calcium imaging. Fluo 8 AM was applied as a fluorescent probe for [Ca^2+^]i detected with green fluorescence. Represented line charts (**b**, **d**) and analysis (**f**, **g**) demonstrate the fluctuation of [Ca^2+^]i following ATP or TG induction. Represented images (**a**) demonstrate the fluctuation of [Ca^2+^]i represent SOCE in three check points including before extracellular calsium induction, reaching the peak level and becoming stable after extracellular calsium induction. Summarized line charts (**c**, **e**) and analysis (**h**, **i**) demonstrate the change of SOCE. Calcium depletion and SOCE were calculated as F1 (peak level of fluorescence)/F0 (initial level of fluorescence). Results were presented as mean ± SD. All datasets were analyzed using one-way analysis of variance followed by Tukey’s post hoc test to compare the differential significance between each two groups. *P < 0.05 vs control, ^∆^P < 0.05 vs 5H_2_O_2_ group was considered statistical significance

FLSs were treated with diverse dose of resveratrol with or without 5 μM H_2_O_2_ for 48 h. Then fluorescent intensity of intracellular calcium ([Ca^2+^]i) was detected via calcium imaging. TG and ATP were used to induce ER store depletion once the curve of the fluorescent intensity was detected stable enough. After TG or ATP induction, all groups present similar shape of curves which have rapid increase and then fall to normal level after surge. Exact line chart analysis demonstrated no significance were observed between diverse group of resveratrol pretreatment (Fig. 1b, d, f, g). When the curve of [Ca^2+^]i became stable again, cells were treated with 2.5 mM CaCL_2_ within the chamber to induce SOCE. However, after extracellular Ca^2+^ injection, FLSs with higher dose of resveratrol presented suppressive effect of SOCE with reduced peak level of fluorescent intensity (Fig. 1a, c, e, h, i). Besides, this kind of effects were observed in partial dose dependent manner of resveratrol.

### Resveratrol reduces ATP or TG induced SOCE via targeting ORAI1–STIM1 complex in FLSs

**Fig. 2:**
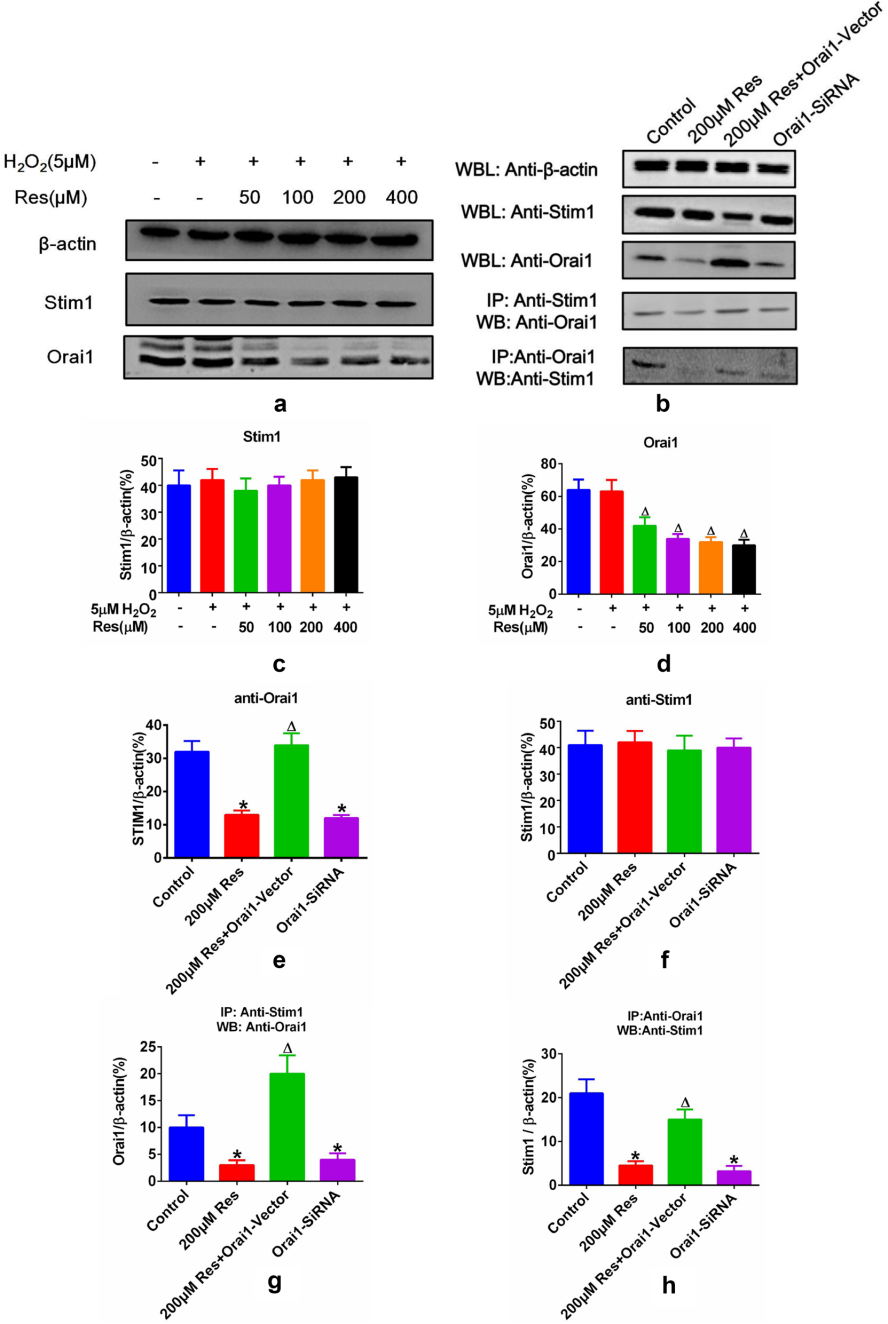
Resveratrol reduces ATP or TG induced SOCE via targeting ORAI1–STIM1 dimer in FLSs. FLSs were treated with diverse dose of resveratrol (50, 100, 200, 400 μM) within 5 μM H_2_O_2_. **a** STIM1 and ORAI1, two essential mediator for SOCE, were detected via western blotting. Represent histograms (**c**, **d**) demonstrate the analytical results of their expressive level following resveratrol treatment. ORAI1 SiRNA and ORAI1 overexpressed vector were respectively applied to knock down or upregulate ORAI1 expression. Co-IP was utilized to acess the inter-relationship between ORAI1 and STIM1. **b** FLSs were undergone diverse treatments including 200 μM resveratrol, 200 μm resveratrol + ORAI1 overexpressive vector and ORAI1 SiRNA and respectively subjected to immunoprecipitation with ORAI1 or STIM1 antibody. Western blot was applied to detect the expression of STIM1 and ORAI1 inside Co-IP (IP) or non-IP (WBL) samples. **e**–**h** Represent histograms demonstrate the exact analytical results.All datasets were analyzed using one-way analysis of variance followed by Tukey’s post hoc test to compare the differential significance between each two groups. Results were presented as mean ± SD and *P < 0.05 vs control, ^∆^P < 0.05 vs 200 μM Res group was considered statistically significance

**Fig. 3:**
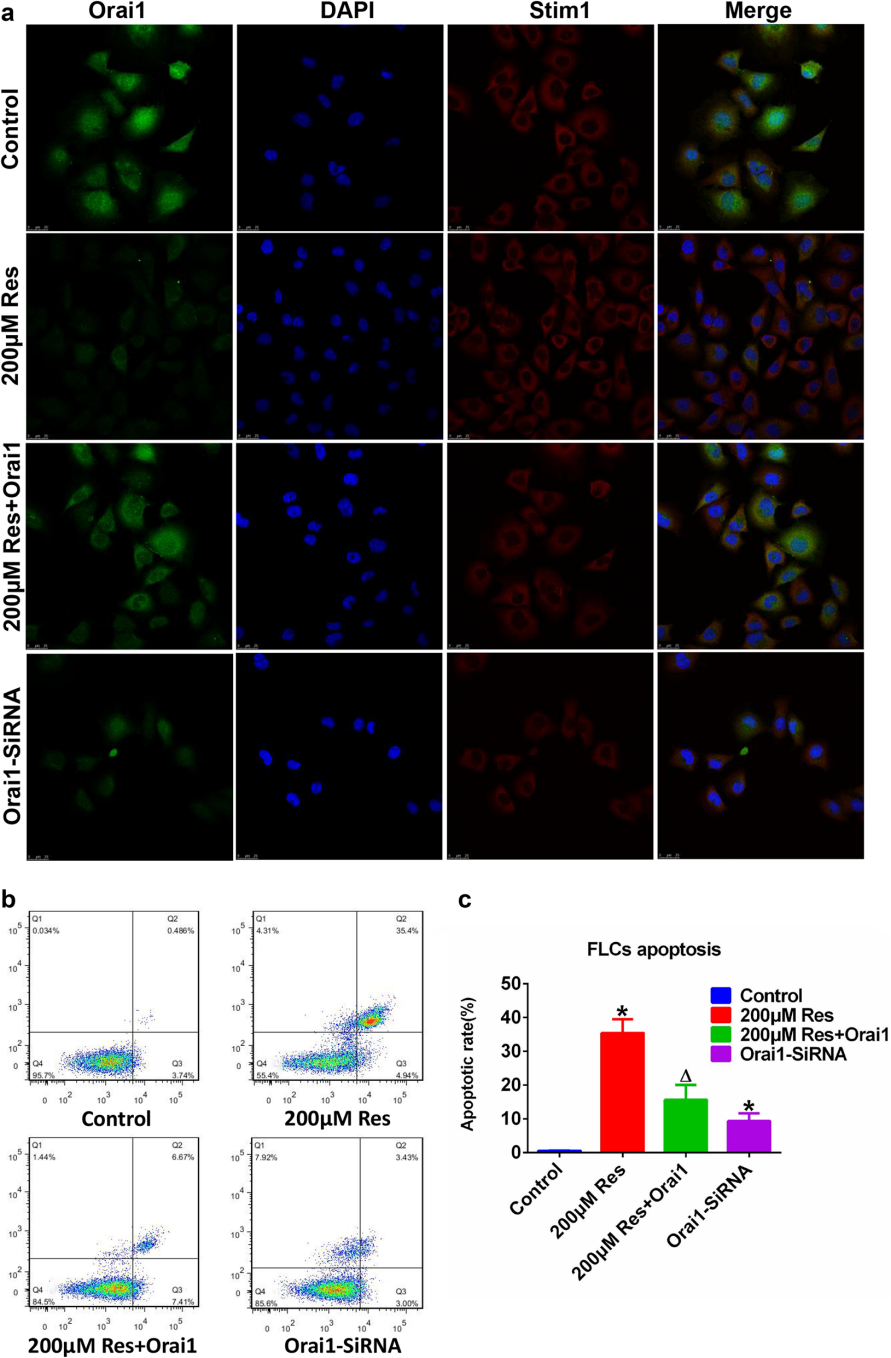
ORAI 1 overexpression restores resveratrol induced apoptosis in FLSs. **a** FLSs were respectively treated with 200 μM Resveratrol, 200 μM Resveratrol + ORAI1-vector and ORAI1 SiRNA. Expressions and distribution of STIM1and ORAI1 were checked via immunofluorescence. ORAI1 and STIM1 were respectively labeled with green and red fluorescence via fluorescent secondary antibody of diverse species matched to primary antibody for STIM1 and ORAI1. DAPI were utilized to conduct nucleus dying with blue fluorescence. Merged pictures demonstrate the overlay among ORAI1, STIM1 and nucleus. **b** Apoptotic rate of FLSs receiving 200 μM Resveratrol, 200 μM Resveratrol + ORAI1-vector and ORAI1 SiRNA were checked via Annex V FITC/PI double staining method and detected via flow cytometry. The meanings of each quadrant are as follows: Lower left: normal cells; upper left: necrotic cells; lower right: early apoptotic cells; upper right: late apoptotic cells. **c** The proportion of apoptotic cells after corresponding treatment was illustrated as the combined percentage of both early and late apoptotic cells. Results were presented as mean ± SD and *P < 0.05 vs control, ^∆^P < 0.05 vs 200 μM Res group was considered statistically significance. All datasets were analyzed using one-way analysis of variance followed by Tukey’s post hoc test to compare the differential significance between each two groups

FLSs were treated with diverse dose of resveratrol with or without 5 μM H_2_O_2_ for 48 h. Then FLSs were lysed and conduct immunoblotting. Two essential mediators of SOCE, STIM1 and ORAI1,were detected via western blotting. After resveratrol incubation, expression level of STIM1 was not affected while ORAI1 expression was evidently suppressed (Fig. 2a, c, d). Besides, this suppressive effect of ORAI1 was observed with dose dependent manner via pre-treatment of resveratrol. Since ORAI1 and STIM1 form complex to play its role in mediating SOCE, Co-IP assay was applied to study the effects of resveratrol on STIM1–ORAI1 complex. ORAI1 overexpressed vector and ORAI1-SiRNA were used to conduct overexpression and down regulation of ORAI1. After Co-IP detection, results showed that resveratrol could interrupt the formation of ORAI1–STIM1 complex which may explain the suppressive effect of SOCE via resveratrol. Besides, ORAI1 overexpression following resveratrol treatment suppressed the interruption. Only treatment of ORAI1-siRNA exerted similar effects as resveratrol (Fig. 2b, e–h). Further confirmation was presented via immunofluorescence assay and subsequent results were consistent with WB and Co-IP results further confirming this biological change and high quality of both overexpressed vector and ORAI1 SiRNA (Fig. 3a).

### ORAI1 overexpression restores resveratrol induced SOCE reduction following ATP or TG induction and resveratrol induced apoptosis in FLSs

**Fig. 4:**
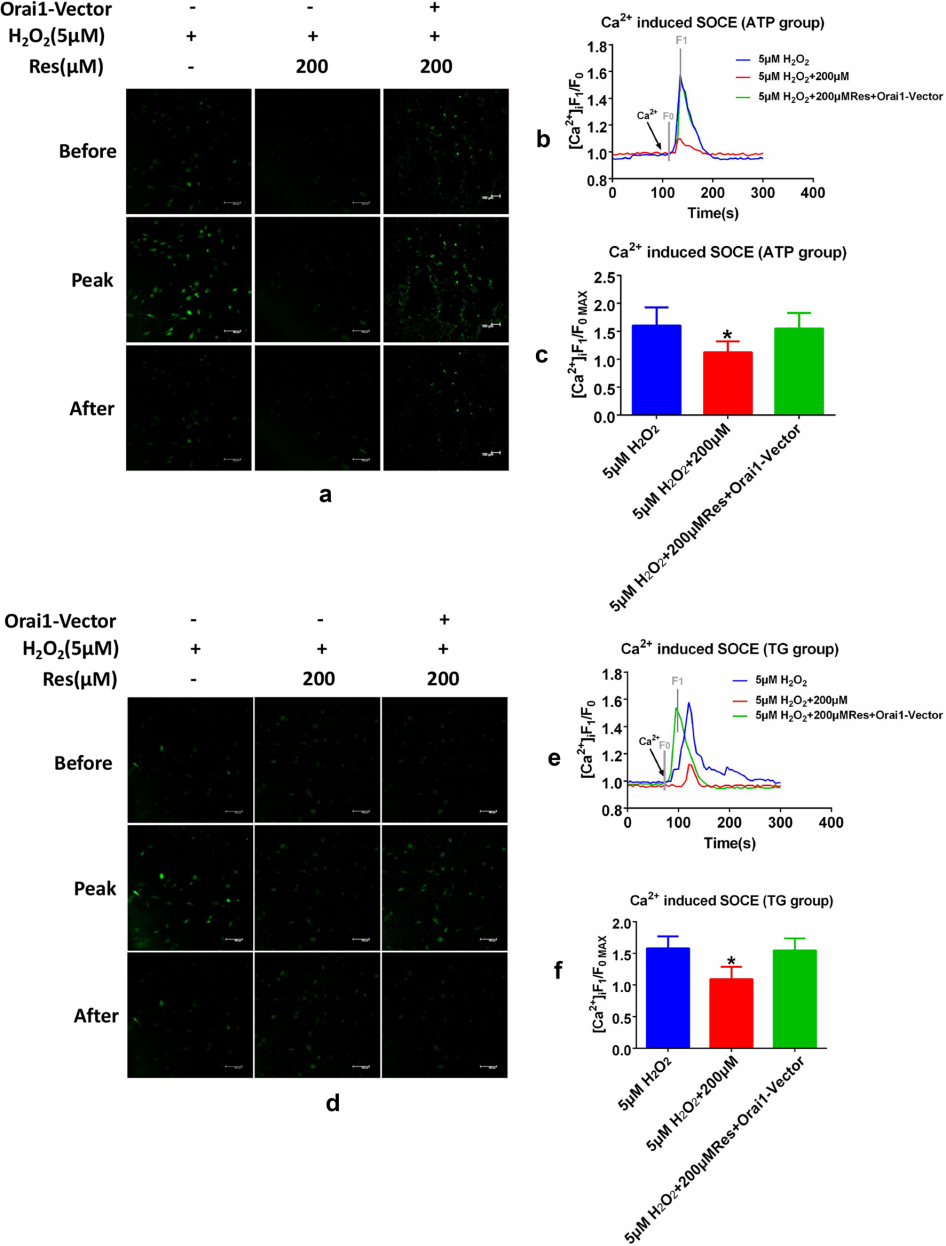
ORAI 1 overexpression restores resveratrol induced SOCE reduction following ATP or TG induction in FLSs. FLSs were respectively treated with 200 μM resveratrol single or coupled with ORAI1-vector within 5 μM H_2_O_2_. Both TG or ATP were utilized to induce calcium store depletion and the following adding of extracellular Ca^2+^ was used to trigger SOCE in FLSs. The real time fluctuation of [Ca^2+^]i was recorded via calcium imagine through confocal laszer stanning microscopy. Represented images (**a**) demonstrate the fluctuation of [Ca^2+^]i represent SOCE following ATP induced ER store depletion in three check point including before extracellular Ca^2+^ induction, reaching the peak level and becoming stable after induction. Summarized lincharts (**b**) and analysis (**c**) demonstrate the change of SOCE. Represented images (**d**) demonstrate the fluctuation of [Ca^2+^]i represent SOCE following TG induced ER store depletion in three check point including before extracellular Ca^2+^ induction, reaching the peak level and becoming stable after induction. Summarized lincharts (**e**) and analysis (**f**) demonstrate the change of SOCE. SOCE were calculated as F1(peak level of fluorescence)/F0(initial level of fluorescence). Results were presented as mean ± SD. All datasets were analyzed using one-way analysis of variance followed by Tukey’s post hoc test to compare the differential significance between each two groups. *P < 0.05 vs control was considered statistically significant

Based on our findings, overexpressed ORAI1 may reverse the SOCE suppression induced via resveratrol. In order to verify this, FLSs were treated with 200 μM resveratrol, which exert potent suppressive effect on SOCE in FLSs compared with other concentration, only or with ORAI1-vector within 5 μM H_2_O_2_ for 48 h. Both ATP or TG induced intracellular ER calcium store depletion and extracellular calcium induced SOCE were detected via calcium imaging. Represented images and traces indicate that no difference was observed in ER calcium store depletion among all groups and SOCE reduction induced via 200 μM resveratrol as the former results. However, compared with single treatment of 200 μM resveratrol, treatment with both 200 μM resveratrol and ORAI1 vector present recovery of SOCE which indicate that ORAI1 overexpression restore resveratrol induced SOCE reduction following ATP or TG induction in FLSs (Fig. 4a–f). Since reduced SOCE and ORAI1 was tightly associated with cell apoptosis, we hypothesize that ORAI1 overexpression can alleviate the apoptosis induced via resveratrol [5]. Annexin V FITC/PE assay was used to detect cell apoptosis and flow cytometry analysis indicate that ORAI1 overexpression could partly recover the apoptotic rate compared with 200 μM resveratrol treatment only in FLSs (Fig. 3b, c).

### Overexpression and suppression of ORAI 1 affect the intensity of adjuvant arthritis following resveratrol administration in rats model

**Fig. 5:**
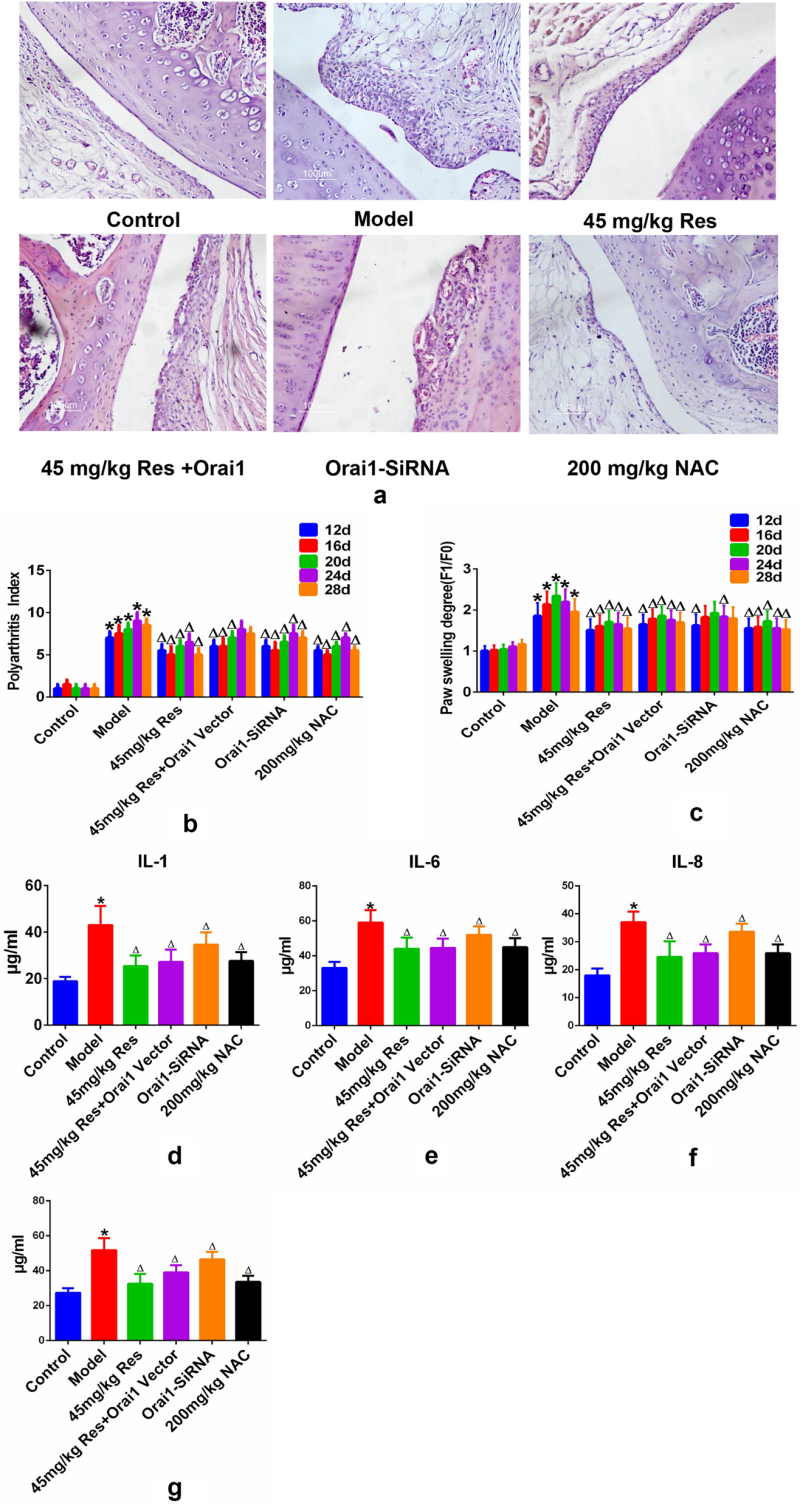
Overexpression and suppression of ORAI 1 affect the severity of adjuvant arthritis following resveratrol administration in rats model. 150 μl Freund’s complete adjuvant was injected into the left hind toe of male SD rats for 20 days to build AA model group while control groups were treated with 150 μl physiological saline in the same parts. All the animals were divided into 6 groups including control group, AA model group and other 4 drug intervention groups in which remaning model rats were respectively treated with 45 mg/kg, 45 mg/kg resveratrol + ORAI-1 overexpressive vector, ORAI-1 SiRNA and 200 mg/kg NAC for 12 days by intragastric administration and joint cavity microinjection with 10 rats/group. Polyarthritis index (**b**) and paw swelling degree (**c**) were recorded every 4 days during the drug-treating process. The Polyarthritis index was used to evaluate secondary lesions in each group of rats. Before the modeling procedure, the animal volume detector was applied to detect the affected hind paw volume of each rat (F0). From the onset of inflammation, the left hind paw volume was measured every 4 days (F1) and the paw swelling degree of the affected paw equal to F1/F0. All the mice were sacrificed in 12 days after treatment mentioned above. (**a**) The limbs were sectioned for histological studies. Whole blood samples were extracted via eye ball removal assay and serum were obtained via centrifugation and restored at − 80 °C. **d**–**h** Represent histogram demonstrate the serum level of IL-1, IL-6, IL-8, IL-10 and TNF-α were detected via ELISA assay. Results were presented as mean ± SD. All datasets were analyzed using one-way analysis of variance followed by Tukey’s post hoc test to compare the differential significance between each two groups. *P < 0.05 vs control and ^∆^P < 0.05 vs Model group were considered statistically significant

It is previously reported that resveratrol-treated for 12 days could reduce paw swelling and arthritis scores at macro level and attenuated inflammatory cell infiltration and synovial hyperplasia in AA rats [20]. Therefore we thought that ORAI1 overexpression following resveratrol treatment may minimize the therapeutic effects of resveratrol on AA and ORAI1 downregulation may have similar effects as resveratrol in alleviating AA. In order to confirm our hypothesis, ORAI1 overexpressed vector and ORAI1-SiRNA were coated inside the liposome and injected into model joint in AA rats. Paw swelling degree and arthritis score were evaluated every 4 days. After animal experiment, all rats were sacrificed and joint were made into pathological slide. HE staining results indicate that ORAI1 overexpression following resveratrol treatment may minimize the therapeutic effects of resveratrol on inflammatory cell infiltration, synovial hyperplasia and necrotic tissue accumulation inside the joint cavity, and ORAI1 downregulation may have similar effects as resveratrol in alleviating AA but the effects were not evident (Fig. 5a). Paw swelling and arthritis scores evaluation was consistent with HE staining and the effects was not evident as well (Fig. 5b, c). Besides, immunohistochemical analysis of Orai1 demonstrated that resveratrol evidently reduced Orai1 expression and treatment of Orai1-vector or Orai-SiRNA also present their desired effects, which further identify the results of HE section, paw swelling and arthritis scores (Additional file 1: Figure S1).

### Overexpression and suppression of ORAI 1 affect the serum inflammatory cytokine level and routine blood index following resveratrol administration in rats model

Resveratrol was reported to alleviate inflammatory injury by various studies. Therefore, we hypothesize that ORAI1 overexpression following resveratrol treatment may suppress this anti-inflammatory effects and ORAI1 knock out may have similar effects as resveratrol. Therefore we detect the serum level of inflammatory cytokines including IL-1, IL-6, IL-8 and TNF-α. Results indicate that resveratrol reduced the level of IL-1, IL-6, IL-8 and TNF-α. ORAI1 overexpression following resveratrol treatment could partly increase the level of IL-1, IL-6, IL-8 and TNF-α, which indicate that ORAI1 overexpression could neutralize the anti-inflammatory effect of resveratrol (Fig. 5d–g). In addition, ORAI1 suppression could exert similar effects as resveratrol both in the aspect of anti-inflammatory capacity indicating that ORAI1 knocked out may have the potential to replace the resveratrol in alleviating AA.

Since inflammatory cytokine paradigms were tightly correlated with blood cells, normal routine blood index was checked (Table 1). Compared with the normal group, the number of WBC, NEUT, LYMPH and MONO in the AA group increased significantly but the PLT did not increase. At the same time, MCV and MCH decreased significantly, indicating that rats had chronic anemia. Reticulocyte elevation suggests compensatory enhancement of hematopoietic function. ORAI1 overexpression can attenuate the therapeutic effects of resveratrol, up-regulating WBC, NEUT, LYMPH, MONO and reduce both MCV and MCH but the effect is not obvious. After down-regulation of ORAI1, it can reduce the number of WBC, NEUT, LYMPH, MONO while elevate both MCV and MCH though the effect is not as obvious as resveratrol. Besides, the other indicators are not significantly different from the normal group, suggesting that knocking out ORAI1 alone can alleviate AA to some extent.

**Table 1:** The blood routine results of grouped rats (n = 6) 150 μl Freund’s complete adjuvant was injected into the left hind toe of male SD rats for 20 days to build AA model group while control groups were treated with 150 μl physiological saline in the same parts

Names	Units	Control	Model	45 mg Res	45 mg Res+Orai1	Orai1-SiRNA	200 mg/kg NAC
Red blood cell (RBC)	10^12^/L	6.94 ± 1.02	7.16 ± 0.94	7.03 ± 1.03	6.94 ± 1.12	7.11 ± 1.03	7.31 ± 0.97
Hemoglobin (HGB)	g/L	146 ± 7.6	149 ± 8.9	142 ± 6.4	156 ± 11.2	153 ± 9.8	152 ± 10.5
Mean corpuscular hemoglobin (MCH)	pg	21.4 ± 0.59	18.9 ± 0.49*	20.3 ± 0.42^**#**^	19.3 ± 0.56^▼^	19.1 ± 0.48^#^	19.2 ± 0.46*
Mean corpuscular hemoglobin concentration (MCHC)	g/L	331 ± 8.92	325 ± 8.72	334 ± 9.23	332 ± 9.12	328 ± 8.92	327 ± 9.35
Hematocrit (HCT)	%	46 ± 2.68	45 ± 2.74	44 ± 3.02	47 ± 2.93	43 ± 2.82	45 ± 2.72
Mean corpuscular volume (MCV)	fL	66.12 ± 1.2	61.28 ± 1.02*	64.31 ± 1.62^#^	63.45 ± 1.03^▼^	62.58 ± 1.03^#^	65.32 ± 1.49*
Platelet (PLT)	10^9^/L	858.23 ± 256.62	764.32 ± 264.54	792.64 ± 278.38	821.61 ± 248.78	785.23 ± 345.65	862.67 ± 292.82
Mean platelet volume (MPV)	fL	8.15 ± 1.45	7.96 ± 1.62	7.45 ± 0.98	7.64 ± 1.03	8.35 ± 1.14	8.27 ± 1.23
Thrombocytocrit (PCT)	%	0.62 ± 0.08	0.58 ± 0.06	0.54 ± 0.07	0.65 ± 0.12	0.61 ± 0.09	0.58 ± 0.11
Platelet distribution width (PDW)	%	8.64 ± 1.23	9.45 ± 1.64	8.67 ± 1.15	9.26 ± 1.65	8.94 ± 1.98	8.92 ± 1.52
Large platelet ratio (P-LCR)	%	11.75 ± 2.23	9.11 ± 1.98	9.85 ± 2.78	11.26 ± 1.84	10.54 ± 2.12	11.56 ± 2.36
White blood cell (WBC)	10^9^/L	12.65 ± 1.62	18.44 ± 1.54*	14.3 ± 1.78^#^	16.5 ± 1.23^▲^	18.1 ± 0.98^#^	13.4 ± 1.56*
Neutrophil (NEUT)	10^9^/L	1.41 ± 0.27	5.28 ± 1.36*	2.20 ± 0.58^#^	3.06 ± 0.56^▲^	2.27 ± 0.32^#^	1.80 ± 0.24*
Percentage of neutrophils	(%)	11.2 ± 2.92	28.65 ± 4.68*	15.4 ± 2.65^#^	18.56 ± 3.46^▲^	12.56 ± 2.42^#^	13.5 ± 2.65*
Lymphocyte (LYMPH)	10^9^/L	8.12 ± 1.78	15.54 ± 2.02*	10.04 ± 1.89^#^	12.78±2.02^▲^	14.73±2.56^#^	9.18±1.52*
Percentage of lymphocyte	%	64.26±4.23	84.32±5.56*	70.23±4.59^#^	77.5 ± 5.21^▲^	81.4 ± 4.21^#^	68.56 ± 5.96*
Monocyte (MONO)	10^9^/L	0.62 ± 0.07	0.96 ± 0.12*	0.67 ± 0.08^#^	0.75 ± 0.07^▲^	0.89 ± 0.11^#^	0.72 ± 0.06*
Percentage of monocyte	%	4.90 ± 0.98	5.20 ± 1.02*	4.68 ± 0.95^#^	4.54 ± 1.06^▲^	4.91 ± 0.85^#^	5.37 ± 1.05*
Eosinophils (EO)	10^9^/L	0.08 ± 0.01	0.1 ± 0.02	0.09 ± 0.01	0.11 ± 0.03	0.11 ± 0.02	0.09 ± 0.03
Percentage of eosinophils	%	0.63 ± 0.12	0.54 ± 0.16	0.62 ± 0.13	0.66 ± 0.14	0.60 ± 0.09	0.67 ± 0.11

All the animals were divided into 6 groups including control group, AA model group and other 4 drug intervention groups in which remaining model rats were respectively treated with 45 mg/kg, 45 mg/kg resveratrol + ORAI-1 overexpressed vector, ORAI-1 SiRNA and 200 mg/kg NAC for 12 days by intragastric administration and joint cavity microinjection with 10 rats/group. whole blood samples were extracted via eye ball removal assay and immediately received blood routine test. Results were presented as mean±SD. All datasets were analyzed using one-way analysis of variance followed by Tukey's post hoc test to compare the differential significance between each two groups

*P < 0.05, vs Control group; ^#^P < 0.05, vs Model group; ^▼^P < 0.05, vs 45 mg Res group (down regulation); ^▲^P < 0.05, vs 45 mg Res group (up regulation)

## Discussion

Taken together, this study addressed that resveratrol reduces store-operated Ca^2+^ entry and enhance the apoptosis of fibroblast-like synoviocytes in adjuvant arthritis rats model via targeting ORAI1/STIM1 dimer. In the present study, resveratrol was found to reduce SOCE following ATP or TG induced calcium store depletion in FLSs. TG is an inhibitor of the endoplasmic reticulum Ca^2+^-ATPase, which prevents the intracellular calcium from flowing into the ER by blocking SERCA, leading to a one-way efflux of calcium from the ER to the cytosol [21]. The efflux of calcium is then excreted out and the addition of extracellular calcium could induce SOCE via SOCs. ATP binds to the purinergic receptor (P2Y or P2X) on the cell membrane, and then couples with the PLC and activates the IP3 receptor on the ER, which leads to emptying of the calcium pool and then the addition of extracellular calcium induces SOCE [22].

Calcium imaging results showed that resveratrol treatment could not affect ATP or TG-induced calcium depletion, indirectly indicating that resveratrol itself does not affect the blocking effect of TG on SERCA and the expression of SERCA itself, and does not affect IP3 activity as well as the expression of P2Y, of which the exact biological process need further validation. Few studies have reported the influences of resveratrol on SERCA activity, IP3-induced calcium release and routine calcium leakage of ER, which is consistent with our results. On this basis, research on SOCE can exclude other influences. Many studies have shown that resveratrol can inhibit SOCE, but its mechanism is diverse [19, 23, 24]. Our research showed that suppressed SOCE was correlated with ORAI1 loss and reduction of ORAI1–STIM1 dimer without expressive difference for STIM1 in FLSs. Resveratrol has been reported to promote autophagy in prostate cancer cells by down-regulating STIM1 and down-regulating SOCE, but the expression of ORAI1 and TRPC1 was not affected during this process [24]. It has been reported that resveratrol can inhibit the binding of STIM1 to ORAI1 by inhibiting the phosphorylation of STIM1 in HEK 293T cells lead to down-regulating of SOCE, which was inconsistent with our results [23]. There are also some reports that resveratrol has no effect on SOCE or extensive inhibition of STIM1 and ORAI1, and we believe that these differences may be related to differences in tissue cells and experimental conditions.

The downregulation of ORAI1 by resveratrol was an interesting finding and our results also showed that ORAI 1 overexpression restore resveratrol induced SOCE reduction following ATP or TG stimulation. This further demonstrates that ORAI, rather than STIM1, plays a role in the resveratrol induced downregulation of SOCE. In addition, after resveratrol treatment, ORAI 1 overexpression restores resveratrol induced apoptosis in FLSs. The mechanism by which resveratrol induces apoptosis is diverse, and ORAI1 may be just one of many targets in its action. We originally thought that the possibility of recovery from apoptosis by reversing resveratrol-induced ORAI1 downregulation was minimal, but the results showed that overexpression of ORAI1 restored resveratrol-induced apoptosis to a certain extent, and the effect was more obvious than expected. This unexpectedly demonstrates that ORAI1 may be a key target for resveratrol to regulate apoptosis in fibroblasts. To further verify the true effect of ORAI1, we packaged ORAI1-SiRNA and ORAI1 overexpressed vectors in liposomes and resuspended in PBS and injected the mixture into the rat lesion using a microinjector. Results showed that overexpression of ORAI1 following resveratrol treatment partly diminish the therapeutic effects of AA by resveratrol while single ORAI 1 knockout present resveratrol-like alleviation on severity of AA.

However, the impact of this treatment was not obvious at the animal level. The reason may be related to the following points. First, the lesions of RA are far more than the changes of fibroblastic cells, and various immune cells, vascular endothelium, fibers and stromal cells are also involved in the process. Resveratrol does not necessarily regulate the SOCs of these cells in the same pattern. Second, the efficiency of this injectable drug delivery method is extremely limited, and there may be not enough carriers that actually play a regulatory role. Therefore, in order to further explore the role of resveratrol at the animal level, the knockout and overexpression mouse model is a more ideal research model.

Overexpression of ORAI1 following resveratrol treatment slightly recovered the therapeutic effects of resveratrol in the serum level of inflammatory marker and oxidative injury while single ORAI1 knockout present resveratrol-like influence on serology test in rats model. Compared with histological analysis, serological effects were more evident, but still not enough to reverse the effects of resveratrol. It also illustrates the diversity of the mechanism of resveratrol in the treatment of AA. Simply reversing the expression of ORAI1 is far from enough to regulate the whole process, which was significantly different from the cell test. The AA rat model had been reported to have abnormal hemogram including elevation of white blood cells, percentage of neutrophils, lymphocytes and monocytes. Besides AA rats model also had anemia and a decrease in MCV and MCH, which is consistent with human RA [25].

This may because under the action of chemokines, monocytes, lymphocytes and neutrophils in the blood swim out of blood vessels and gather at the site of inflammation to release oxygen free radicals, participating in the development of AA. At the same time, IL-1, IL-6 and TNF-α can inhibit bone marrow erythroid hematopoiesis, promote the apoptosis of bone marrow erythroid precursor cells, reduce the secretion of erythropoietin and promote iron metabolism abnormalities [26–28]. Reticulocyte erythrocytosis suggests compensatory enhancement of hematopoietic function [29]. Overexpression of ORAI1 following resveratrol treatment partly diminished the effects of hemogram via resveratrol and single ORAI1 suppression slightly reduced WBC, NEU% and elevated both MCV and MCH compared with model group.

This is because resveratrol is a natural anti-inflammatory and anti-oxidant polyphenol and it has been proven to have the function of relieving abnormal blood picture of AA in the early stage [2]. Its effect of treating chronic anemia was reported to be related to inhibition of IL-1, IL-6 and TNF-α, which was consistent with our results [30, 31]. However, what is the mechanism under the action of resveratrol on blood cells, whether it can affect the proliferation, apoptosis, differentiation and signal transduction of immune cells needed to be confirmed. We found that ORAI1 may be a key target for resveratrol to regulate apoptosis in fibroblasts, and whether ORAI1 is a target for resveratrol to regulate immune cells in the course of RA is unknown. Because the sample acquisition of synovial cells in RA patients is very difficult, this paper only validates the related effects of resveratrol at the animal and cell test levels. AA cannot completely simulate the characteristics of human RA, so it has certain limitations. Therefore, RA clinical trials and cell mechanism studies are needed in the future to elucidate the regulation of resveratrol on SOCs. The verification of this role should be extended to a wider variety of cell research including immune cells, blood cells and so on. This is due to the multi-cell involvement in RA.

## Conclusion

**Fig. 6:**
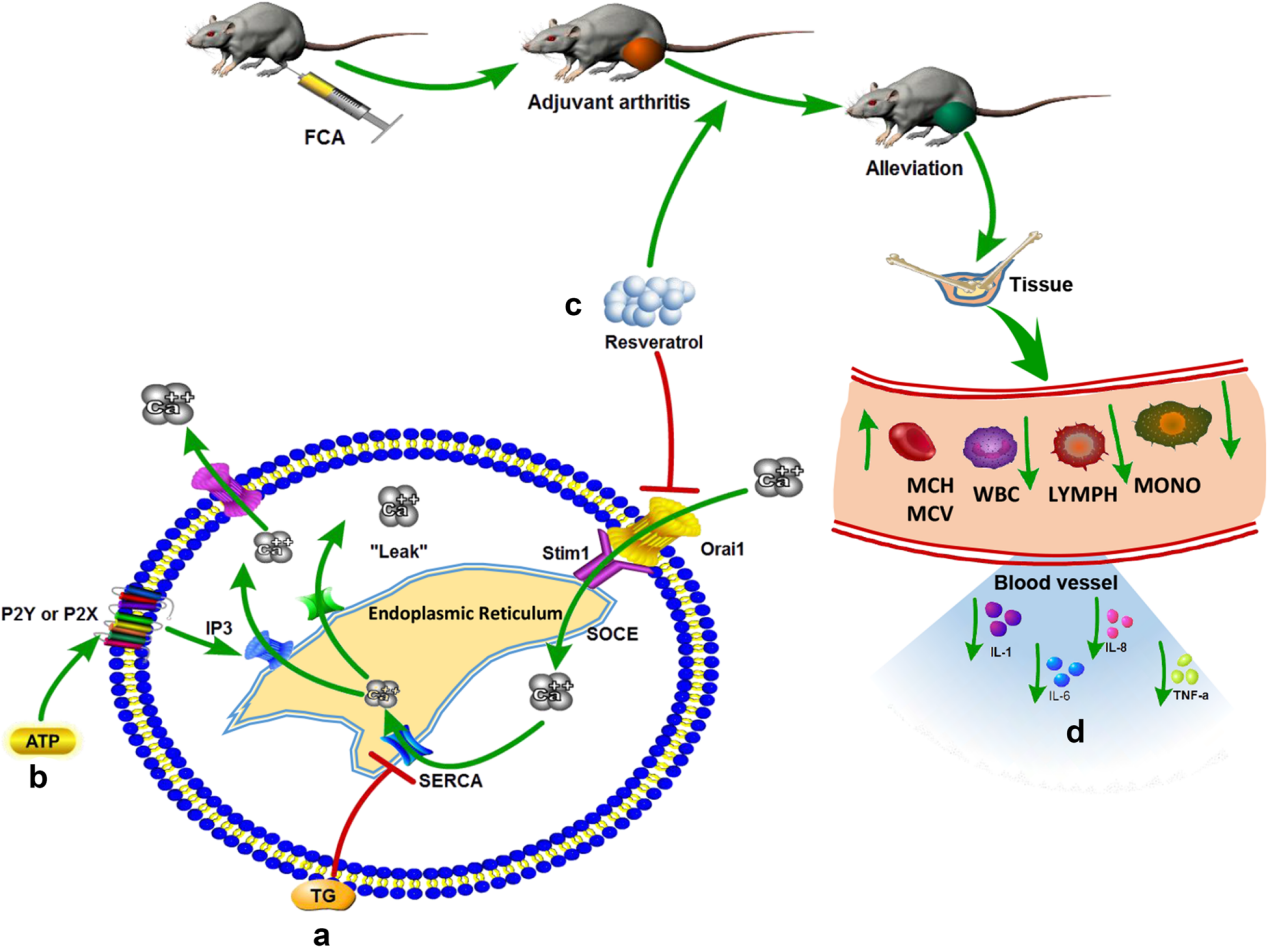
Graphic abstract for the functional role of resveratrol on adjuvant arthrits. **a** TG, an inhibitor of the endoplasmic reticulum Ca^2+^-ATPase, prevents the intracellular calcium from flowing into the ER by blocking SERCA, leading to an one-way efflux of calcium from the ER to the cytosol. **b** ATP binds to the purinergic receptor (P2Y or P2X) on the cell membrane, and then couples with the PLC and activates the IP3 receptor on the ER leading to emptying of the calcium pool, at which time the addition of extracellular calcium induces SOCE. **c** In the present study, resveratrol reduces TG or ATP induced store-operated Ca^2+^ entry and enhances the apoptosis of fibroblast-like synoviocytes in adjuvant arthritis rats model via targeting ORAI1/STIM1 dimer. **d** Alleviation of tissue injury via resveratrol in AA rats was accompanied with reduced inflammatory level manifested as reduced IL-1, IL-6, IL-8 and TNF-α, and improved hemogram with reduced level of WBC, NEUT, LYMPH, MONO and increased MCV and MCH

In summary, this study addressed that resveratrol reduces store-operated Ca^2+^ entry and enhance the apoptosis of fibroblast-like synoviocytes in adjuvant arthritis rats model via targeting ORAI1/STIM1 dimer (Fig. 6). This study explains to some extent the potential regulatory targets and regulation of SOCE in the treatment of AA, providing a preliminary theoretical basis of future accurate treatment of RA based on ORAI targeted interruption.

## Materials and methods

### ORAI-1 overexpressed vector and ORAI-1 SiRNA construction

SiRNA (5′-AGUUCUUACCGCUCAAGAGGCAGGC-3′) targeting the rats ORAI1 transcription and a non-silence sequence (control) were designed and constructed into a pU6-mRFP expression vector as described elsewhere [32]. The ORAI1 cDNA sequence was constructed into a pCMVPuro01 expression vector (Sinogen, China). The forward and reverse primer sequences for vector building were 5′-CTA GTC TAG AAT GCA TCC GGA GCC CGC CC 3′ and 5′-TCC TTC GAA CTA GGC ATA GTG GCT GCC GG 3′ respectively. Empty vectors were transfected to FLSs as a negative control [33].

### Animal modeling and drug treatment

Male Sprague-Dawley (SD) rats (8 weeks, 200–250 g) were obtained from the animal center of Anhui Medical University (Hefei, China) and all animal experiments were conducted with the consent of medical ethics committee in Anhui Medical University. 150 μl Freund’s complete adjuvant (FCA, Sigma, USA) was injected into the left hind toe of male SD rats for 20 days while control groups were treated with 150 μl physiological saline in the same parts [3]. All the animals were divided into 6 groups including control group, adjuvant-arthritis model group and other 4 drug intervention groups in which model rats were respectively treated with 45 mg/kg resveratrol, 45 mg/kg resveratrol + ORAI1 overexpressive vector, ORAI1 SiRNA and 200 mg/kg *N*-acetyl-l-cysteine (NAC, Sigma, USA) for 12 days by intragastric administration or joint cavity microinjection. All the mice were sacrificed in 12 days after treatment mentioned above. Polyarthritis index and paw swelling degree were recorded every 4 days during the drug-treating process. The Polyarthritis index was used to evaluate secondary lesions in each group of rats. Polyarthritis indexes for each foot: 0 points = normal; 1 point = erythema and slight swelling of the ankle joint; 2 points = erythema extended from the ankle to palm or toe joint combined with slight swelling; 3 points = erythema extended from the ankle to palm or toe joint combined with moderate swelling; 4 points = erythema extended from the ankle to palm or toe joint combined with severe swelling (Up to 12 points per rat). The paw swelling degree is also used to evaluate the severity of the lesion in the affected limb. Before the inflammation, the animal volume detector (Yanyi Biotechnology, Jinan, China) was applied to detect the affected hind paw volume of each rat (F_0_). From the onset of inflammation, the left hind paw volume was measured every 4 days (F_1_) and the paw swelling degree of the affected paw equals to F_1_/ F_0_.

### Cell culture, isolation and transfection

Low-passage FLSs were cultured in DMEM medium and maintained at 37 °C in a 5% CO_2_ atmosphere waiting for transfection. The day before transfection, trypsinize and count the cells. Plate 4 × 10^4^ cells per well in 0.5 ml of complete growth medium to make cell density between 50–80% on the day of transfection. For each well, dilute 100 pmol ORAI1 overexpressive vector or ORAI1 SiRNA into 100 μl of Opti-MEM I Reduced Serum Medium (Invitrogen, USA) without serum and dilute 5 μl of Lipofectamine 3000 (Invitrogen, USA) into the above diluted ORAI1 overexpressive vector or ORAI1 SiRNA solution, mix gently and incubate for 25 min at room temperature to form Lipofectamine complexes. Remove growth medium from cells and replace with 0.5 ml of complete growth medium. Add 100 μl of the Lipofectamine complexes directly to each well and mix gently. Remain complexes and incubate the cells at 37 °C in a CO_2_ incubator for 18–24 h post-transfection before following experiments.

### [Ca^2+^] measurement

Intracellular Ca^2+^ measurement was performed as previous described [34]. Simply, FLSs were seeded on circular coverslips and incubated with 10 μmol/L Fluo-8 (invitrogen, USA) combined with 0.02% pluronic acid F-127 at 37 °C for 20 min. Then the cells were washed via preheated Ca^2+^-free PBS (37 °C) for 3 times, then loaded on a chamber with 500 μL Ca^2+^-free PBS that contained 140 mmol/L NaCl, 5 mmol/L KCl, 1 mmol/L MgCl_2_, 10 mmol/L glucose, 0.2 mmol/L EGTA, and 5 mmol/L HEPES (pH 7.4) and transferred to platform under microscope. Ca^2+^ release was triggered via treating FLSs with 4 μmol/L TG or 100 μmol/L ATP for 5 min. The real-time fluctuation of fluorescence representing [Ca^2+^]i was recorded by Leica confocal laser scanning microscopy (model LEICA.SP5-DMI6000-DIC; Leica Microsystems GmbH) with a 488 nm excitation and 515 nm long pass emission wavelength. Briefly, before triggering Ca^2+^ release, recording area of each Fluo-8 labeled cells within the view were traced and then Ca^2+^ release was triggered via treating FLSs with 4 μmol/L TG or 100 μmol/L ATP within the chamber and wait for 5 min to observe the overall changing process of fluorescence. Once the fluorescent intensity becomes stable, calcium influx was initiated via 2.5 mM extracelluar calcium and the fluctuation of fluorescence was recorded until the baseline became stable again. Variation in [Ca^2+^]i were presented as the ratio of subsequent fluorescence relative to the intensity before the administration of TG, ATP or Calcium. The magnitude of calicum release and SOCE were represented as the ratio of peak fluorescence relative to the intensity before the administration of TG, ATP or Calcium (F1/F0). Fluorescence intensity were collected on the average of 20-30 cells for each data. All the analysis were performed on Leica LAS AF lite 2.6.0 software and details were described else where (https://www.jove.com/video/56317).

### Immunofluorescence

Immunofluorescence was conducted to detect the expression and location of ORAI1 and STIM1. Seed 1 × 10^5^ digested cells on coverslips and incubate the cells for 24 h to maintain a stable growing state in 35 mm dishes containing aforementioned culture medium. Then grouped cells on the coverslips were transfected with diverse vector or SiRNA according to the experimental requirements and incubate the cells for 24–48 h. Next, coverslips were taken out to wash off the remaining liquid. Wash the coverslips with PBS twice for 5 min, the cells were fixed with 4% paraformaldehyde for 10 min and permeabilized in 0.3% triton-X for 5 min. Then wash the coverslips twice again and the cells were blocked in 1% bovine serum albumin (BSA) for 30 min and incubated at room temperature for 20 to 30 min. After that, adding the first antibody (Cell signaling, USA) of ORAI1 (1:200, rabbit) and STIM1 (1:250, mouse) diluted in PBS together inside new 35 mm dishes and incubated the cells overnight at 4 °C under darkness. Then wash the coverslips twice and incubate the cells with second antibody (Alexa Fluor 488 and Alexa Fluor 594) according to the species of first antibody. After incubation, wash the coverslips twice again and incubate DAPI 5ug/ml diluted in PBS for 5 min to label the nuclei. Then wash the coverslips twice again. Images were analyzed under a Leica confocal laser scanning microscopy (model LEICA.SP5-DMI6000-DIC; Leica Microsystems GmbH). ORAI1 (green) was observed with a 495 nm excitation and 519 nm long pass emission wavelength and STIM1 (red) was observed with a 591 nm excitation and 614 nm long pass emission wavelength. Merged picture showed the overlay between STIM1 and ORAI1 inside the cell.

### Western blot analysis

Western blot analysis was performed as previously described in detail [20]. Simply, cells were lysed and centrifugated at 12,000*g* for 10 min at 4 °C. The supernatants were loaded in each well and subjected to 10% SDS-polyacrylamide gel electrophoresis (SDS-PAGE) and transferred to PVDF membrane (Millipore, USA). Next, 5% nonfat milk was used to block the PVDF membrane in washing buffer for 2 h at room temperature and then incubated with primary antibody including STIM1, ORAI1 and β-actin (Biosharp, China) with diverse diluted ratio (1:1500, 1:2000 and 1:10,000) at 4 °C overnight. On the following day, washing PVDF membrane with TBST-T and incubate with 1:10,000 dilution of horseradish peroxidase HRP-labeled anti-rabbit IgG (Beyotime, China) for 1 h at room temperature. Finally each protein bands were visualized using enhanced chemiluminescence reagents (BOSTER, Wuhan, China).

### Immunoprecipitation

Protein extracted from FLSs were mixed with IP lysis buffer(contains protease inhibitor) and incubated at 4 °C for 30 min and then centrifuged at 12,000×*g* for 30 min. 1 μg corresponding antibody for STIM1 or ORAI1 and 10–50 μl protein A/G-beads (ThermoFisher, USA) were added into the supernatants and incubated on shaking table at 4 °C overnight. After the immunoprecipitation, the mixtures were centrifuged at 3000*g* at 4 °C for 5 min and remove the supernatants. Wash the protein A/G-beads with lysis buffer twice. Finally, add 15 μl of 2× SDS buffer and boil for 10 min. Proteins were then subjected to 10% SDS-PAGE and transferred to membranes, probed with antibodies against the interacting protein of interest, and processed for Western blotting as described above.

### HE staining and immunohistochemistry

After corresponding animal experiment, rats were sacrificed via cervical dislocation. Knee-joint was extracted and fixed in 4% paraformaldehyde. Then tissues were dehydrated in ethanol and finally embedded in paraffin. 5 mm-thick histologic cuts from the paraffin blocks were obtained and stained with hematoxylin-eosin (HE) for general histology. Immunohistochemistry for Orai1 (1:50; Ab59330, Abcam), was performed after antigen retrievals in citrate buffer. Samples were incubated with a secondary antibody (anti-rabbit IgG antibody, Jackson ImmunoResearch) and mounted with mounting media. Orai1 was stained as brown particles. The images of the stained tissue were captured via a light microscope.

### Blood index test

2 ml blood samples were collected after eyeball extraction and divided into 2 EDTA-2Na tubes and one of them was immediately centrifuged to get plasma with the speed of 4000 rpm and stored at − 20 °C, and the other one was instantly conducted blood routine test. Blood routine test was conducted via automatic blood cell analyzer (Mindry BC5310, China). Serum inflammatory cytokines including IL-1, IL-6, IL-8, IL-10 and TNF-α were detected via ELISA kits (Abcam, USA) according to the instruction. Read the absorbance on a microplate reader for aforementioned cytokines at a wavelength of 450 nm. Calculate the mean value of triplicate readings for each sample or standard.

### Statistical analysis

Data were analyzed by Graphpad PrismV7.04 and SPSS 24.0 software. The results were expressed as mean ± SD unless otherwise noted. All experiments were repeated at least three times and all datasets were analyzed using one-way analysis of variance followed by Tukey’s post hoc test to compare the differential significance between each two groups and a P value < 0.05 were used to determine statistical significance.

## Additional file

**Additional file 1: Figure S1.** 150 μl Freund’s complete adjuvant was injected into the left hind toe of male SD rats for 20 days to build AA model group while control groups were treated with 150 μl physiological saline in the same parts. All the animals were divided into 6 groups including control group, AA model group and other 4 drug intervention groups in which remaining model rats were respectively treated with 45 mg/kg, 45 mg/kg resveratrol + ORAI-1 overexpressive vector, ORAI-1 SiRNA and 200 mg/kg NAC for 12 days by intragastric administration and joint cavity microinjection with 10 rats/group. All the mice were sacrificed in 12 days after treatment mentioned above. (a) The limbs was sectioned for immunohistochemical analysis of Orai1 expression.

## Data Availability

All the data support the present study are available from corresponding author on reasonable request.

## Abbreviations

SOCE: store operated calcium entry
SOCs: store operated calcium channels
TG: thapsigargin
ATP: adenosine triphosphate
SiRNA: small interfering RNA
FLSs: fibroblast like synoviacytes
STIM1: stromal interaction molecule 1
ORAI1: calcium release-activated calcium channel protein 1
