# Supplementary figures and images for: Resveratrol reduces store-operated Ca2+ entry and enhances the apoptosis of fibroblast-like synoviocytes in adjuvant arthritis rats model via targeting ORAI1–STIM1 complex
Source: Biol Res. 2019 Aug 19;52:45. doi: 10.1186/s40659-019-0250-7 (PMC6699118; doi:10.1186/s40659-019-0250-7)

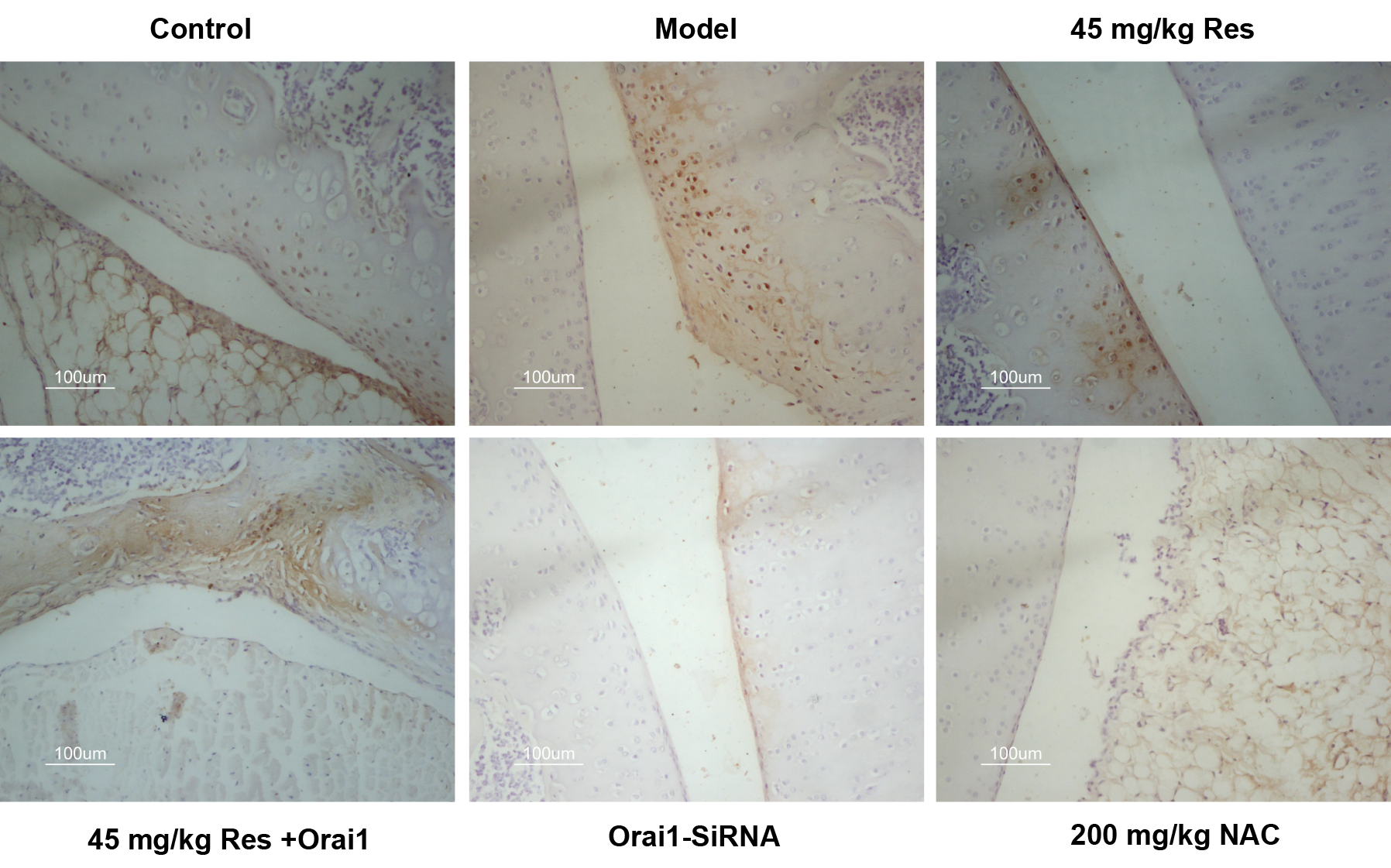

Supplement: Supplementary file 1 — Additional file 1: Figure S1. 150 μl Freund’s complete adjuvant was injected into the left hind toe of male SD rats for 20 days to build AA model group while control groups were treated with 150 μl physiological saline in the same parts. All the animals were divided into 6 groups including control group, AA model group and other 4 drug intervention groups in which remaining model rats were respectively treated with 45 mg/kg, 45 mg/kg resveratrol + ORAI-1 overexpressive vector, ORAI-1 SiRNA and 200 mg/kg NAC for 12 days by intragastric administration and joint cavity microinjection with 10 rats/group. All the mice were sacrificed in 12 days after treatment mentioned above. (a) The limbs was sectioned for immunohistochemical analysis of Orai1 expression. [file 40659_2019_250_MOESM1_ESM.tif]
